# A Run of Bad Cases

**Published:** 1885-10

**Authors:** 

**Affiliations:** Montgomery, Texas; Montgomery, Texas


### A RUN OF BAD CASES.

### ECLAMPSIA AND PLACENTA-PRÆVIA.

*By Drs. Irion and Cochran, Montgomery, Texas.*

For Daniels' Texas Medical Journal.

WE would like to know if there are among the many readers of your Journal any other two M. D.'s as unfortunate as we have been lately, not as to results, but in meeting with such a number of bad cases in such a short time? Within the last four months we have had three cases of puerperal eclampsia, and three of placenta prævia.

As there has been much discussion in the profession as to the use of the lancet in puerperal convulsions, we will give our plan of treatment, and let the result speak for itself. Right here we will say that, so as far bleeding for convulsions is concerned, the senior member of this firm has broken the point of his lancet, and the junior member never had one.

Case 1. Dr. I. was called to Mrs. A., white, primipara, age 20 years. He found her in a terrific convulsion, during which the child was born. The first thing done was to give hypodermically one half grain morphia sulp., then took away the placenta, and administered per os:

| | |
|---|---|
| Tinc. Aconite | m. v. |
| Fl. Ext. Gelsem | ʒss. |
| Chloral Hyd. | gr. xxx. |

The patient being in an unconcious condition, he had to introduce a spoon between the teeth and hold the nose until the medicine was swallowed. This treatment controlled the spasms for about eight hours, when a slight recurrence of them took place, but was promptly relieved by a repetition of the first prescription, with a complete recovery, the child and mother both doing well.

Case 2. Mrs. B., mulatto, primipara, aged 16 1-2 years. Dr. C. was called, and found the patient blind, unconcious, and having had two convulsions. The same general plan of treatment as in case No. 1, with the addition of ergot to bring on labor. In about two hours spasms returned, but were promptly relieved as before. In about twelve hours more both Drs. I. and C. were called, and found labor commencing. By means of Puzo's plan of treatment with injections of hot water to neck of the womb, labor was hurried through to a successful termination, and patient was thought to be all right, but owing to the ignorance and negligence of attendants, we learned, she afterwards died.

Case 3. Mrs. T., white, primipara, age 19 years. Dr. I. was called, and found she had been having convulsions for twelve hours, with no sign of labor. The same treatment as in the other two cases, controling the spasms, until, in about twelve hours, labor commenced, and progressed slowly, and when the head was pressing upon the perinæum, spasms came on again. The former treatment was continued, with chloroform to anæsthesia and labor still continuing slowly, upon Dr. C.'s arrival, forceps were applied and child delivered. Unfortunately, however, in the expulsion of the child's head, the mother's perinæum was badly ruptured. As soon as the placenta, clots of blood, etc., were cleared away, the rent in the perinæum was closed with four interrupted sutures, silk being used, as we had no silver wire. The mother and child are both doing well.

We give the three cases mentioned to show our faith in aconite, gelsemium and chloral hydrate to control spasms, in preference to the use of the lancet. Our treatment in the three cases of placenta prævia mentioned, was Miller's modification of Puzo's plan, which consists in artificial dilatation, ergot, hot injections to neck of womb to induce labor, and the tampon to arrest hemorrhage, and irritate the mouth of the womb. As soon as possible we ruptured the membranes, and left the rest to nature. All three cases made a good recovery.

We report these cases to show our success with the plans of treatment adopted, and in hopes they may be of assistance to some brother M. D. in times of trouble. By reporting unusual cases and courses of treatment we may mutually assist each other in the noble work of relieving suffering humanity and preserving life.

